# Central Actions of Leptin Induce an Atrophic Pattern and Improves Heart Function in Lean Normoleptinemic Rats via PPARβ/δ Activation

**DOI:** 10.3390/biom14081028

**Published:** 2024-08-18

**Authors:** Blanca Rubio, Cristina Pintado, Lorena Mazuecos, Marina Benito, Antonio Andrés, Nilda Gallardo

**Affiliations:** 1 Biochemistry Section, Faculty of Sciences and Chemical Technologies, University of Castilla-La Mancha, Avda. Camilo José Cela 10, 13071 Ciudad Real, Spain; blancamaria.rubio@cnic.es (B.R.); lorena.mazuecos@uclm.es (L.M.); 2 Molecular Regulation of Heart Failure Research Group, National Cardiovascular Research Center Carlos III (CNIC), Melchor Fernández Almagro 3, 28029 Madrid, Spain; 3 Biochemistry Section, Faculty of Environmental Sciences and Biochemistry, University of Castilla-La Mancha, Avda. Carlos III s/n, 45071 Toledo, Spain; cristina.pintado@uclm.es; 4 DOE Research Group, Institute of Biomedicine, University of Castilla-La Mancha (IB-UCLM), 13071 Ciudad Real, Spain; 5 ICTS Bioimagen Complutense (BioImaC), Universidad Complutense de Madrid, P°. de Juan XXIII 1, 28040 Madrid, Spain; marben14@ucm.es

**Keywords:** PPARβ/δ, leptin, MRI, cardiac remodeling, Wistar rat

## Abstract

Leptin, acting centrally or peripherally, has complex effects on cardiac remodeling and heart function. We previously reported that central leptin exerts an anti-hypertrophic effect in the heart via cardiac PPARβ/δ activation. Here, we assessed the impact of central leptin administration and PPARβ/δ inhibition on cardiac function. Various cardiac properties, including QRS duration, R wave amplitude, heart rate (HR), ejection fraction (EF), end-diastolic left ventricular mass (EDLVM), end-diastolic volume (EDV), and cardiac output (CO) were analyzed. Central leptin infusion increased cardiac PPARβ/δ protein content and decreased HR, QRS duration, and R wave amplitude. These changes induced by central leptin suggested a decrease in the ventricular wall growth, which was confirmed by MRI. In fact, the EDLVM was reduced by central leptin while increased in rats co-treated with leptin and GSK0660, a selective antagonist of PPARβ/δ activity. In summary, central leptin plays a dual role in cardiac health, potentially leading to ventricular atrophy and improving heart function when PPARβ/δ signaling is intact. The protective effects of leptin are lost by PPARβ/δ inhibition, underscoring the importance of this pathway. These findings highlight the therapeutic potential of targeting leptin and PPARβ/δ pathways to combat cardiac alterations and heart failure, particularly in the context of obesity.

## 1. Introduction

Accumulating evidence indicates that an excess of nutrient intake is harmful to the heart as a result of its role in cardiac remodeling [1,2]. In fact, cardiovascular diseases (CVD) represent a significant global health burden, especially among individuals with obesity and its associated metabolic disturbances, including metabolic syndrome and diabetes. Obesity and insulin resistance are recognized inducers of cardiac hypertrophy. While cardiac hypertrophy, which involves the enlargement of heart muscle cells, is the more commonly discussed form of remodeling, cardiac atrophy is also an important aspect.

Interestingly, multiple studies have shown that an atrophic pattern caused by reduced fuel supply to the heart leads to a lower cardiovascular risk profile. This is characterized by a decreased cardiac workload due to enhanced metabolic efficiency and alterations in signaling pathways associated with cardiac remodeling, potentially improving cardiac function and efficiency [1,3,4]. Although the mechanisms behind cardiac atrophy resulting from metabolic unloading are still not well understood, some researchers have suggested that signals from the intermediary metabolism of energy-providing substrates might play significant roles [1].

Leptin, a hormone predominantly secreted by adipose tissue, is well-known for its role in regulating energy balance by inhibiting hunger, thereby reducing food intake and promoting energy expenditure. Moreover, the circulating levels of leptin correlate positively with body mass index (BMI), reflecting the body’s fat stores. Leptin, acting through the long isoform of the leptin receptor (Ob-Rb) in the hypothalamus, stimulates both thyrotropin-releasing hormone (TRH) and corticotropin-releasing hormone (CRH) secretion by the paraventricular nucleus (PVH), mediating leptin’s stimulation of the sympathetic nervous system (SNS) [5]. Nevertheless, the neural circuitry that integrates leptin and other metabolic signals to control energy homeostasis and body weight remains poorly defined.

Beyond its metabolic functions, leptin has been shown to exert complex effects on the cardiovascular system. Notably, leptin influences cardiac structure and function, supported by studies demonstrating its involvement in both hypertrophic and protective mechanisms within the heart [6,7].

For instance, studies in obese and leptin-deficient mouse models (*ob*/*ob* and *db*/*db*) [8,9] suggest leptin might protect against LV hypertrophy during the progression of obesity. Importantly, it has been demonstrated that chronic intracerebroventricular (ICV) leptin infusion protects the heart from adverse remodeling and cardiac contractile dysfunction after myocardial infarction (MI) [10].

Despite these insights, the overall effects of leptin on cardiac remodeling in humans remain largely unknown. Recently, a cross-sectional study by Kamimura et al. [11] examined the relationship between plasma leptin concentrations and LV structure and function indices in a community-based cohort of 1172 African Americans with preserved EF. The study found sex- and BMI-specific associations between leptin levels and LV mass, suggesting that leptin’s protective effects against LV hypertrophy might be more pronounced in certain subpopulations.

Similarly, PPARβ/δ plays a significant role as a nuclear receptor acting as a nutrient sensor. Activation of PPARβ/δ regulates various inflammatory processes, lipid metabolism, and energy utilization pathways, which collectively enhance cardiac efficiency and protect against metabolic stress [10,11]. Moreover, enhancing PPARβ/δ activity, either through natural means or pharmacological intervention, represents a potential therapeutic approach to mitigate the metabolic complications associated with obesity. Therefore, we propose that PPARβ/δ may interact with leptin signaling pathways, potentially influencing cardiac remodeling and function in response to metabolic stress and nutrient availability.

In fact, recent data from our laboratory support a potential protective role of central leptin signaling against cardiac hypertrophy and provide new insights into the mechanisms by which leptin, acting at a central level, activates PPARβ/δ, harmonizing anti-hypertrophic responses, redox state, proteasome-dependent protein degradation, and autophagy in the heart [12]. Therefore, balancing their activities could help manage obesity and its comorbidities, which often include insulin resistance and cardiovascular diseases. However, the *in vivo* mechanisms underlying the effects of both leptin and PPARβ/δ on cardiac structure and function are not fully understood.

In this study, we focused on investigating the influence of central leptin on heart rate and cardiac output in normoleptinemic rats with normal leptin sensitivity. Specifically, we aimed to establish in vivo the role of PPARβ/δ on cardiac remodeling when the stimulus applied to induce it was the activation of central leptin signaling. To achieve this, we administered the selective PPARβ/δ antagonist GSK0660.

Our findings support the concept that leptin, acting centrally, induces an atrophic pattern in the heart and contribute to a healthier myocardial profile [10,12]. More importantly, our data indicate that administration of the PPARβ/δ antagonist led to a condition resembling left ventricular hypertrophy (LVH) in obesity, characterized by an abnormal increase in left ventricular myocardial volume while still maintaining a preserved ejection fraction. This supports the notion that pharmacological inhibition of PPARβ/δ throughout the body abolishes the central actions of leptin in cardiac remodeling.

## 2. Materials and Methods

### 2.1. Experimental Animals

Experiments were performed on 3-month-old male Wistar rats. All the animals were fed with a standard chow diet and water and maintained in ventilated-controlled quarters (20–25 °C temperature, 50–55% humidity, daily 12-h light cycle 7 a.m.–7 p.m.). Animals were randomly housed individually to control their food intake and thereby avoid differences in adipose tissue weight and serum levels of hormones and metabolites that depend on the amount of feed eaten by animals. Body weight and food intake were monitored daily during all the treatments. Animals were handled according to the European Union’s laws (2010/63/EU) and following Spanish regulations (RD 53/2013) for laboratory animals’ use. The experimental protocols were approved by the Institutional Scientific Ethics Committee under project license es PR-31-2019. All efforts were made to reduce the number of animals used and minimize animal suffering.

### 2.2. Intracerebroventricular Leptin Administration

Leptin infusion was performed as previously described [12,13]. Rats were anesthetized in an induction chamber with 4% isoflurane (0.8 L/min oxygen flow) (Pharmacia-Upjohn, Barcelona, Spain) and then were placed in a stereotaxic frame (David Kopf, Los Angeles, CA, USA) with a thermal blanket below. Leptin or saline (PBS) was administered for 7 days in the lateral ventricle through a cannula connected to an osmotic minipump (Alzet, Palo Alto, CA, USA), with a releasing rate of 1 µL/h and filled with 0.0082 µg/µL (0.2 µg/day) rat leptin (Sigma), or its vehicle (saline). To analyse the effect of central infusion of leptin, rats were treated with either saline (SS, n = 5) or leptin (Lep, n = 5) for 7 days. A third group of rats (PF, n = 5) was treated with saline and *pair-fed* to the amount of food consumed by the leptin-treated group. Body weight and food intake were measured daily during the experiment.

### 2.3. Pharmacological Administration of the PPARβ/δ Antagonist GSK0660

To analyse the contribution of PPARβ/δ, we administer in vivo GSK0660, a selective antagonist inhibitor of PPARβ/δ, to a group of rats at a dose that does not induce toxic side effects but abolished the effects on body weight mediated either by central leptin infusion or by caloric restriction in *pair-fed* rats, as previously described [11,12]. Briefly, GSK0660 was diluted first in DMSO (less than 1%) and later in 0.9% NaCl. Then, it was infused daily intraperitoneally (i.p; 1 mg/kg per day) for 7 days in the PF+GSK0660 (n = 5) and Lep+GSK0660 (n = 5) group of rats. Control groups PF+DMSO (n = 5) and Lep+DMSO (n = 5) received an intraperitoneal injection of the vehicle at 2 mL/kg (0.062% DMSO) in parallel. Seven days after minipump implantation, the animals were fasted overnight. Rats were anesthetized by CO_2_ inhalation and killed by decapitation. Blood was removed and centrifuged (2000× *g*, 15 min), and serum was recovered and frozen in liquid nitrogen at −70 °C until use. Hearts were rapidly excised and washed twice in Henseleit buffer at 37 °C. After removing the major blood vessels and connective tissue, hearts were dried and weighed. Epididymal (eWAT), perirenal (PrWAT), and brown adipose tissue (BAT) were rapidly excised and weighted. The tibia from each animal was dissected, and after removing the muscle, the length of the tibia was measured using a digital micrometer caliper, and the heart weight/tibial length ratio was calculated. Hereafter, atria were removed, and both ventricles were used in all analyses after being flash-frozen in liquid nitrogen and stored at –70 °C until use. The hypothalamic regions were carefully dissected as previously described [14]. After that, hypothalamic regions were frozen in liquid nitrogen and stored at −70 °C until further processing.

### 2.4. Biochemical Assessment

Serum hormone and metabolite levels were measured as described [12,13,15] following the manufacturer’s instructions. Glucose was measured using an Accutrend Glucose Analyser, (#05050472223, Roche, Basel, Switzerland), leptin was quantified using a Rat Elisa Kit (#RD291001200R, Biovendor, Heidelberg, Germany), and insulin was determined using a Rat Elisa Kit (#10-1251-01, Mercodia, Uppsala, Sweden).

### 2.5. Quantitative Transcription Analysis with Real-Time Polymerase Chain Reaction (qRT-PCR)

RNA from the hypothalamus was obtained using All Prep DNA/RNA/Protein Mini kit (Cat. No. 80004, Qiagen, Venlo, The Netherlands) following the manufacturer’s instructions. The cDNA was synthesised from 1.5 µg of DNase-treated RNA. Relative quantification of the long isoform of the leptin receptor (*Ob-Rb*), corticotropin-releasing hormone (*Crh*), and thyrotropin-releasing hormone (*Trh*) mRNA levels was performed by real-time PCR according to the manufacturer’s protocol on an ABI PRISM 7500 FAST Sequence Detection System instrument and software (PE Applied Biosystem, Foster City, CA, USA). To standardize the amount of cDNA added to the reaction, amplification of endogenous control 18S rRNA was included in separate wells using VIC (TaqMan Assay) or primers as a real-time reporter. The ΔΔCT method was used to calculate the relative differences between experimental conditions and control groups as a fold of change in gene expression. Details about the genes used in this study are provided in Supplementary Data Table S1.

### 2.6. Western Blot Analysis

100 mg of frozen ventricles were ground under liquid N_2_ before homogenization (2 mL buffer/g tissue) in Henseleit buffer (1 mM PMSF, 100 mM EDTA, 2 mM Na_3_VO_4_, 10 μg/mL leupeptin, 10 μg/mL aprotinin and 1 μg/mL pepstatin), using a manual Dounce homogenizer, followed centrifugation at 800× *g* for 5 min at 4 °C to produce a total extract. Protein lysates (equal amounts of 50 µg) were separated under reducing conditions (10% polyacrylamide concentration gels) SDS-PAGE. Samples were previously mixed with SDS sample buffer and boiled at 95 °C for 10 min. Proteins were transferred to nitrocellulose sheets (0.2 µm, Bio-Rad, Hercules, CA, USA) and incubated overnight (12–16 h) at 4 °C with the appropriate primary antibodies, followed by incubation at room temperature for 2 h with corresponding secondary antibody conjugated with horseradish peroxidase. Primary polyclonal antibody was anti-PPARβ/δ (1:1000, ab23676) and anti-β-actin (1:1000, ab8226) from Abcam, Cambridge, UK. The secondary antibody used was goat anti-rabbit conjugated with horseradish peroxidase (1:4000, 172-1019) from Bio-Rad, Madrid, Spain. Blots were repeated three times to ensure the reproducibility of the results. The immunocomplexes formed were visualized using the ECL Western-blotting detection kit (Amersham Biosciences, Inc., Piscataway, NJ, USA), and the images were subjected to a densitometric analysis with a G-Box Densitometer. Bands were quantified by scanning densitometry with the exposure in the linear range using Gene Tools software (Syngene, Cambridge, UK). Samples from rats infused with vehicle, leptin, or GSK0660 in all experimental conditions were run on the same gel to allow a direct comparison. β-actin was used as a control for protein loading.

### 2.7. Cardiac Magnetic Resonance Imaging and Analysis

Magnetic Resonance Imaging (MRI) studies were performed in a Bruker Biospec 70/30 scanner using a combination of a linear coil (for transmission) with a cardiac phase array coil (for reception). Animals were anesthetized with isoflurane (3% for induction and 1% for maintenance) and placed in an MRI-adapted stereotaxic holder. Respiration rate and ECG were continuously monitored during the scans (SA Instruments). MRI acquisition protocol included an initial flash sequence to center the Field of View (FOV) and plan the short axis (Table 1). Each short-axis slice (total n = 20) was acquired with an integrated sequence.

**Figure 1:**
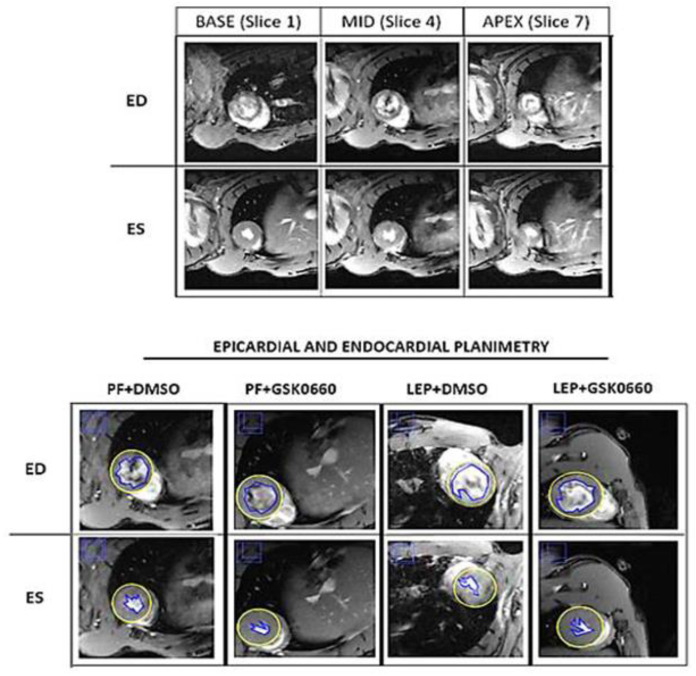
Multiplane assessment of the left ventricular wall by MRI. The figure shows a representative image base to apex (base [base], mid zone [mid] and apex [ape]) short axis end of diastole (End-Diastole, ED) and the end of systole (End-Systole, ES; **upper panel**). Additionally, a representative image of the planimetry of the epicardium (yellow line) and endocardium (blue line) used for the analysis of the four treated groups is shown (**lower panel**).

Cardiac function and morphology were assessed from the cinema sequences using the freely available software Segment v4.0 R11044b (http://segment.heiberg.se, accessed on 29 Jaunary 2024) [16]. The primary outcome measure was the LVEF (left ventricular ejection fraction), determined by cardiac MRI. Segmentation of the left ventricle was performed manually. Papillary muscles were included when defining endo- and epicardial borders. Definitions of end-diastole and end-systole were calculated by the software. Following manual LV segmentation, the software calculated end-diastolic volume (EDV), end-diastolic left ventricular mass (EDLVM), ejection fraction (EF), and cardiac output (CO) automatically [17,18]. Regional wall analyses were performed on a slice-by-slice basis, according to the user manual. In brief, the left ventricle was covered by five short-axis slices (one slice in the base/three slices in the middle/one slice in the apex; Figure 1).

### 2.8. Statistical Analysis

Data are expressed as mean ± SEM. Statistical analysis was performed using the GraphPad Prism version 8.0.2 for Windows (GraphPad Software). Differences between the two groups were assessed using the unpaired Student’s *t*-test. Significant differences between more than two groups were assessed by one-way ANOVA followed by the Tukey test as a post-hoc analysis (different letters indicate significant differences). Specific analysis and symbols used have been specified in each figure legend. A *p* value of ≤0.05 was considered statistically significant. The number of rats used per experiment is stated in each figure legend.

## 3. Results and Discussion

### 3.1. Validation of Central Leptin Infusion and Pharmacological Inhibition of PPARβ/δ Activity

In this work, we infused leptin ICV in 3-month-old Wistar rats for 7 days to stimulate PPARβ/δ activity in the heart and co-administered GSK0660 (i.p) to abolish the effects of this transcription factor in leptin-treated rats. Initially, we wanted to confirm the ability of exogenous leptin to activate hypothalamic leptin signaling through its own receptor by measuring the hypothalamic mRNA levels of the long-form *Ob-Rb* leptin receptor and those of *Crh* and *Trh*, two target genes for the action of leptin in saline, *pair-fed* and leptin-treated rats. As expected, leptin-induced hypothalamic *Ob*-*Rb*, *Crh,* and *Trh* gene expression compared to the saline and *pair-feeding*, indicating that central leptin sensitivity was maintained during the 7 days of leptin treatment and supporting its role in regulating energy balance and metabolic processes (Table 2).

**Figure 2:**
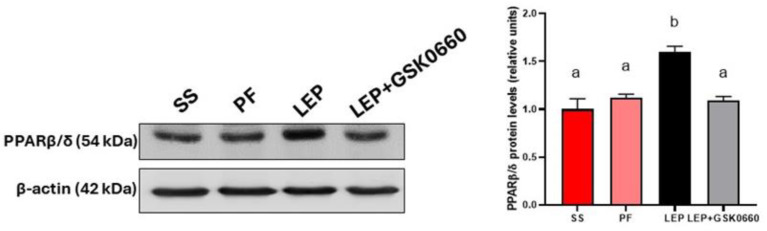
Effect of central leptin on PPARβ/δ protein levels in the heart. Western-Blot analysis of PPARβ/δ in 50 µg of total extracts from cardiac ventricles after central saline or leptin infusion. The values are expressed relative to the SS group. Data are the mean ± SEM (n = 5) per group of animals. Statistical analysis was performed using one-way ANOVA followed by Tukey’s test. Different letters indicate significant differences (*p* ≤ 0.05). Groups: SS: saline-infused rats fed *ad libitum*; PF: saline-infused *pair-fed* rats; Lep: leptin-infused rats; Lep+GSK0660: leptin-infused rats and treated with GSK0660. A representative Red Ponceau staining of the nitrocellulose membrane for total protein normalization prior to immunodetection and the quantitative densitometry readings of PPARβ/δ protein from the Western-Blot analysis are depicted in Figure S1 and Table S2, respectively, in the Supplemental Data.

We next confirmed that chronic central infusion of leptin increased PPARβ/δ protein content in the heart, compared to the saline and *pair-feeding* (Figure 2), highlighting its role in leptin’s cardiac effects. Moreover, the pharmacological inhibition of PPARβ/δ, via the in vivo administration of the selective antagonist GSK0660, blunted the induction of PPARβ/δ in the heart mediated by central leptin (Figure 2).

### 3.2. Effect of Leptin-GSK0660 Co-Treatment on Biological Characteristics of the Rats

**Figure 3:**
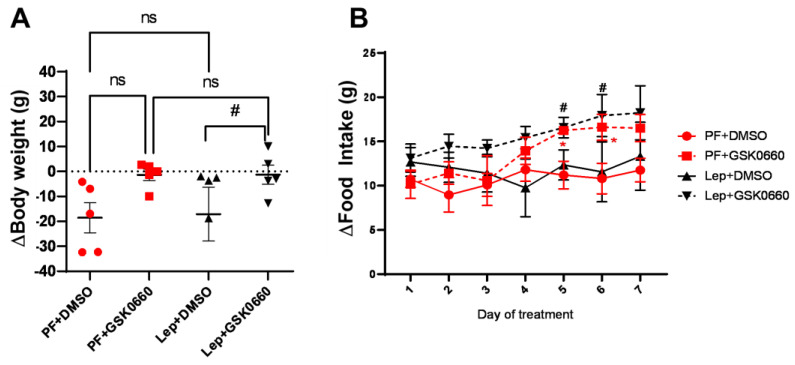
Variation in body weight (**A**) and food intake (**B**) after 7 days of central administration of leptin plus DMSO, leptin plus GSK0660, or vehicle-infused *pair-fed* rats plus DMSO and vehicle-infused *pair-fed* rats plus GSK0660. Data are the mean ± SEM (n = 5) per group of animals. Differences between two groups were assessed using the unpaired Student’s *t*-test (# *p* ≤ 0.05 Lep+GSK0660 vs. Lep+DMSO; * *p* ≤ 0.05 PF+GSK0660 vs. PF+DMSO; Figure 2A,B). Groups: PF+DMSO: vehicle-infused *pair-fed* rats plus DMSO; PF+GSK0660: vehicle-infused pair-fed rats plus GSK0660; Lep+DMSO: leptin plus DMSO; Lep+GSK0660: leptin plus GSK0660.

Confirming previous data [13,15], rats infused with central leptin (Lep+DMSO) did not change significantly their body weight (Figure 3A), average food consumption (Figure 3B), and adiposity (Table 3) compared to *pair-fed* groups infused with vehicle (PF+DMSO). The intraperitoneal (i.p) administration of GSK0660 during 7-day (Lep+GSK0660 and PF+GSK0660) abolished the effects on body weight and food intake (Figure 3A,B) mediated either by central leptin infusion (Lep+GSK0660) or by caloric restriction (PF+GSK0660) in *pair-fed* rats.

Central leptin infusion did not induce changes in serum glucose and leptin levels compared to the pair-fed group infused with vehicle (PF+DMSO) (Table 3), as previously reported [12]. Nevertheless, GSK0660 treatment notably increased serum levels of insulin (Table 3), indicating that GSK0660 induced insulin resistance in 3-month-old Wistar rats.

**Figure 4:**
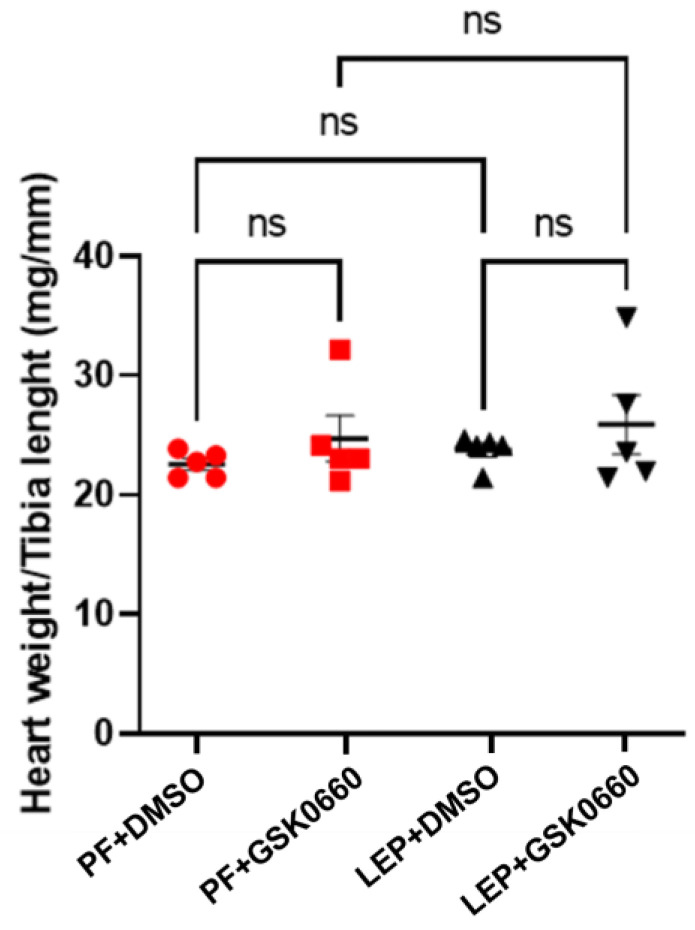
Graphic representation of heart weight/tibia length ratio (mg/mm). Data are the mean ± SEM (n = 5) per group of animals. Differences between the two groups were assessed using the unpaired Student’s *t*-test (*p* ≤ 0.05). Groups: PF+DMSO: vehicle-infused *pair-fed* rats plus DMSO; PF+GSK0660: vehicle-infused *pair-fed* rats plus GSK0660; Lep+DMSO: leptin plus DMSO; Lep+GSK0660: leptin plus GSK0660.

In small animals with rapid changes in body weight, as in rats, the normalization of heart size relative to body weight is not a good indicator of adverse cardiac remodeling development [19]. Instead, as the heart weight/tibia length index remains constant after maturity [19], we used this index to analyze the effects of leptin and GSK0660 on cardiac remodeling. As observed in Figure 4, this index did not show significant differences between rats treated for 7 days with leptin or vehicle, indicating that leptin treatment did not seem to induce a significant alteration of the ventricular wall. However, in the group of animals treated with GSK0660, the ratio was slightly higher compared to its control group treated with DMSO (Figure 4).

### 3.3. Effect of Leptin-GSK0660 Co-Treatment on Cardiac Electrical Properties

To gain a deeper insight into the effects of central leptin on cardiac electrical properties and remodeling, we studied the influence of central leptin treatment in the absence or presence of GSK0660 on cardiac function by analyzing both electrical and physiological patterns.

Left ventricular hypertrophy (LVH) is defined as an increase in left ventricular mass (LVM) associated with structural changes in the myocardium. This occurs in response to mechanical and/or neurohormonal stimuli, leading to an increased workload on the myocyte, which, under biomechanical stress, can induce hypertrophy development [20].

It has previously been reported that ventricular wall thickening is associated with changes in cardiac depolarization and repolarization states, manifested as altered patterns in the duration and amplitude of the QRS complex, as well as QT interval prolongation [21,22]. Accordingly, we used this approach to assess cardiac function and electrical activity in our experiments.

Indeed, as presented in Table 4, we compare the mean values of QRS duration, R wave amplitude as a representation of the QRS complex voltage, and corrected QT duration (QTc) at baseline (t0) and final time points of the treatments (t7) to underscore physiological changes or other alterations in cardiac structure.

The QRS duration and R wave amplitude in lead II voltages, at different times, showed a reduction in the group of animals treated with central leptin, being significant only for the R wave amplitude measurement. These changes suggest a decrease in abnormal ventricular wall growth, which could be indicative of a more atrophic pattern. This is in consonance with previous studies performed in rodents [10,12]. Interestingly, these differences were not observed in the group of animals treated with the PPARβ/δ antagonist. By comparison, QTc duration only increased in the group of animals treated with the PPARβ/δ antagonist when comparing baseline (t0) and final time (t7), indicating a potential alteration in ventricular repolarization, which can have implications for arrhythmogenesis and electrical instability.

In fact, the longer QRS duration and higher R wave voltage are used as measures of relative sensitivity and specificity of abnormal ventricular wall growth, which, in combination with other physiological parameters, facilitate the detection of left ventricular hypertrophy. The results presented in this work indicate that rats treated with the PPARβ/δ antagonist present an altered ECG, possibly due to increased ventricular wall thickness, contrasting with the characteristic atrophic pattern observed in rats treated centrally with leptin, suggesting that the effects observed with central leptin treatment are mediated by leptin-specific pathways rather than PPARβ/δ signaling.

Given that PPARβ/δ has been implicated in the regulation of ion channels and cellular processes involved in cardiac repolarization [23,24,25], antagonism of PPARβ/δ may disrupt these processes, leading to QT prolongation.

Moreover, previous studies have demonstrated the role of disturbed PPARβ/δ activity in the development of metabolic syndrome and its components, including insulin resistance [26], which are associated with QT interval prolongation and increased cardiovascular risk [25,27,28,29].

On the other hand, it is known that leptin signaling improves insulin sensitivity, which is important for changing QTc duration and potentially reducing arrhythmogenic risk [30,31]. Improved insulin sensitivity through effective leptin signaling can lead to better regulation of glucose and lipid metabolism, thereby stabilizing cardiac electrophysiology and reducing the likelihood of QTc prolongation and related arrhythmias.

In addition to these measures facilitating screening of early-stage pathology development (QRS duration, R amplitude, and long QTc), an increase in heart rate (HR) can be used as an indicator of poor prognosis. Various studies associate increased mortality with an elevated heart rate, finding a positive correlation between the two. Similarly, some authors suggest that reducing HR could be used as a potential target for treating patients with heart failure [32].

As seen in Table 5, animals treated with central leptin were able to reduce heart rate, which was not observed in animals additionally treated with the PPARβ/δ antagonist. In the GSK0660-treated groups (PF+GSK0660 and Lep+GSK0660), an increase in heart rate was induced compared to their controls (PF+DMSO and Lep+DMSO). Surprisingly, a significant increase in R-R interval duration (ms) occurred in *pair-fed* control animals treated with the vehicle (PF+DMSO).

This result indicates that central leptin signaling may act as a regulator against the development of cardiac arrhythmias, probably due to its ability to impact cardiomyocyte size [33,34], improve insulin sensitivity, and modulate the autonomic nervous system activity and the expression of ion channels involved in cardiac repolarization [30,31]. Interestingly, Lin et al. [35] have suggested that circulating leptin directly modulates cardiac electrical properties, including heart rate and QT interval, via its receptors expressed in cardiac tissue. These findings suggest a mechanism by which leptin may influence cardiovascular function independently of the central pathways, indicating the potential implications of dysregulated leptin signaling in cardiovascular disorders, including the development of bradycardia, QT interval prolongation, and ventricular arrhythmias.

Therefore, while the precise role of leptin in protecting against cardiac arrhythmias is still being elucidated, our data suggests that central leptin actions may have beneficial effects on cardiac electrophysiology and rhythm regulation

### 3.4. Effect of Leptin-GSK0660 Co-Treatment on Cardiac Remodeling and Function

**Figure 5:**
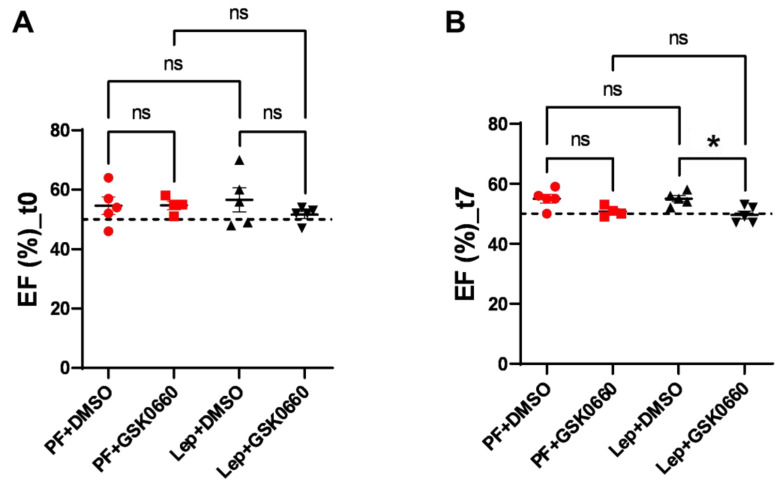
EF values (%) obtained after analysis of the MRI images with the Segment program at t0 (**A**) and t7 (**B**) times. Values are expressed relative to control rats (PF+DMSO). Results are the mean ± SEM (n = 5) per group of animals. One-way ANOVA followed by Tukey test (*p* ≤ 0.05). Differences between treatments were assessed using the unpaired Student’s *t*-test (* *p* ≤ 0.05) Lep+GSK0660 vs. Lep+DMSO. Groups: PF+DMSO: vehicle-infused pair-fed rats plus DMSO; PF+GSK0660: vehicle-infused pair-fed rats plus GSK0660; Lep+DMSO: leptin plus DMSO; Lep+GSK0660: leptin plus GSK0660.

Next, we evaluated alterations in cardiac function using Magnetic Resonance Imaging (MRI) to monitor the response to the progression of central leptin or vehicle treatment. As expected, at baseline (t0), there were no differences in ejection fraction (EF, %) between groups (Figure 5). However, a decrease in EF was induced at the final time (t7) in the Lep+GSK0660 group, which was statistically significant compared to its control group (Lep+DMSO). This decrease only fell below the 50% threshold when the PPARβ/δ activity was inhibited in the central leptin-treated group (Lep+GSK0660). Although EF% values are considered borderline between 41 and 49% [36,37,38], this should not be considered an indicator of heart failure development; instead, it indicates a loss of contractile capacity.

Studies in animal models and some human studies have shown that high-fat diets can lead to cardiac dysfunction, including reduced ejection fraction, as evidenced [39,40]. These effects appear to be mediated by various mechanisms, including inflammation, oxidative stress, mitochondrial dysfunction, and insulin resistance, all of which can impair cardiac contractility and function. While these studies primarily focus on insulin resistance and mitochondrial dynamics in heart disease, there could be implications for the role of leptin signaling in cardiac function. Importantly, vehicle-treated and centrally leptin-treated groups showed preserved ejection fraction after 7 days of treatment (Figure 5).

**Figure 6:**
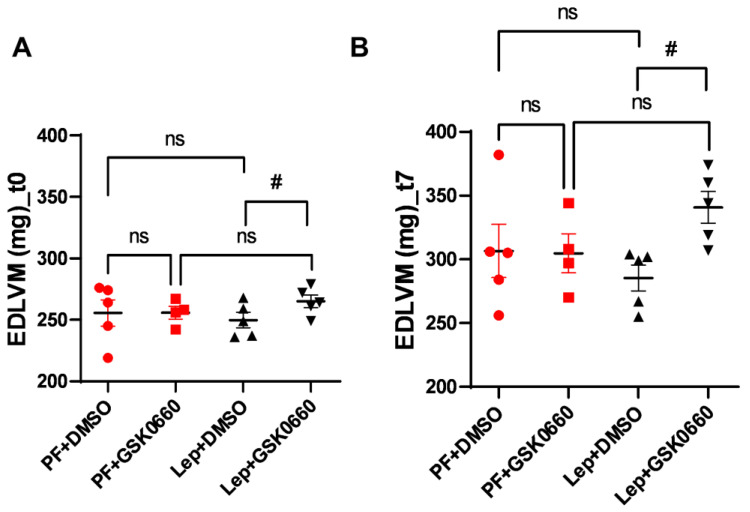
EDLVM values, expressed in gr, obtained after analysis of the MRI images with the Segment program at t0 (**A**) and t7 (**B**) times. Values are expressed relative to the control group (PF+DMSO). Results are the mean ± SEM (n = 5) per group of animals. One-way ANOVA followed by Tukey test (*p* ≤ 0.05). Differences between treatments were assessed using the unpaired Student’s *t*-test (# *p* ≤ 0.05) Lep+GSK0660 vs. Lep+DMSO. Groups: PF+DMSO: vehicle-infused *pair-fed* rats plus DMSO; PF+GSK0660: vehicle-infused *pair-fed* rats plus GSK0660; Lep+DMSO: leptin plus DMSO; Lep+GSK0660: leptin plus GSK0660.

As already mentioned, analysis of MRI allows for a more effective estimation of variations in ventricular mass. We observed that animals treated with the vehicle did not present differences in end-diastolic left ventricular mass (EDLVM) (Figure 6) at baseline and post-treatment times. However, in animals treated centrally with leptin, EDLVM was significantly greater in the group where PPARβ/δ action was blocked using GSK0660. Surprisingly, these animals already showed differences in ventricular mass at baseline. These differences increased at the final time (t7), with EDLVM being significantly greater in this group (Lep+GSK0660).

Thus, this study supports that central leptin treatment at a low dose (0.2 µg/day) promotes an atrophic pattern in the myocardium and that PPARβ/δ is required for leptin signaling to remodel cardiac tissue in rats with normal leptin sensitivity.

**Figure 7:**
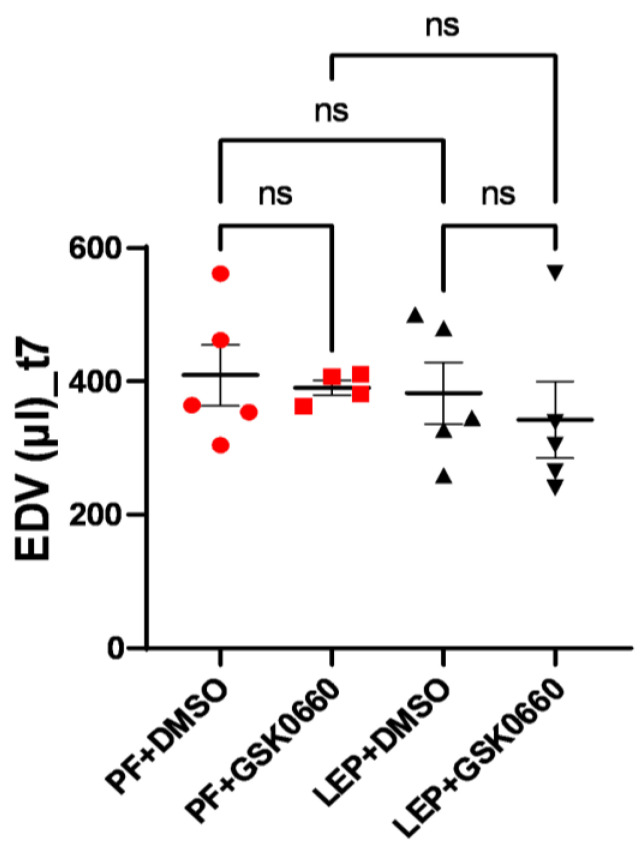
EDV values at t7, expressed in μL, obtained after analysis of the MRI images with the Segment program. Values are expressed relative to the control group (PF+DMSO). Results are the mean ± SEM (n = 5) per group of animals. One-way ANOVA followed by Tukey test (*p* ≤ 0.05). Groups: PF+DMSO: vehicle-infused *pair-fed* rats plus DMSO; PF+GSK0660: vehicle-infused *pair-fed* rats plus GSK0660; Lep+DMSO: leptin plus DMSO; Lep+GSK0660: leptin plus GSK0660.

To measure the potential impact of increased ventricular mass on ventricular filling volume, end-diastolic volume (EDV) was measured at the final time (t7). Although differences were not significant, a slight decrease in final ventricular filling volume was observed for the group of animals with dual treatment (ICV-Leptin and IP-GSK0660) (Figure 7).

**Figure 8:**
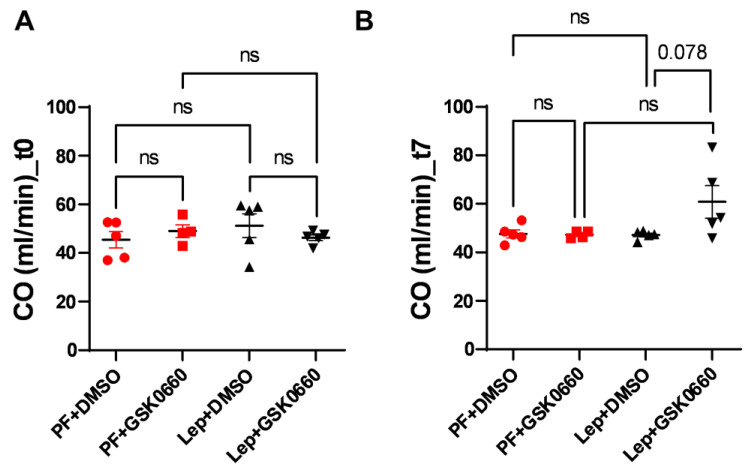
CO values (mL/min) obtained after analysis of the MRI images with the Segment program at t0 (**A**) and t7 (**B**) times. Values are expressed relative to the control group (PF+DMSO). Results are the mean ± SEM (n = 5) per group of animals. One-way ANOVA followed by Tukey test (*p* ≤ 0.05). Differences between treatments were assessed using the unpaired Student’s *t*-test (*p* ≤ 0.05). Groups: PF+DMSO: vehicle-infused *pair-fed* rats plus DMSO; PF+GSK0660: vehicle-infused *pair-fed* rats plus GSK0660; Lep+DMSO: leptin plus DMSO; Lep+GSK0660: leptin plus GSK0660.

Finally, possible changes in cardiac output (CO) were studied. As seen in Figure 8, the differences in ventricular mass did not induce changes in CO pre- and post-treatment, despite an increase in the Lep+GSK0660 group (t7) being observed, which was not significant (*p* = 0.078). The lack of significant changes in CO may be attributed, at least in part, to compensatory mechanisms that were initially induced during the development of hypertrophy to prevent an increase in CO [41]. These compensatory mechanisms could involve various physiological adaptations aimed at maintaining cardiac output despite changes in ventricular mass. One such mechanism could involve an increase in heart rate (HR), which was observed in the animal treated with leptin and the PPARβ/δ antagonist (Lep+GSK0660), as depicted in Figure 8.

Finally, supporting the cardioprotective effects of central leptin reported herein in normoleptinemic adult rats with normal leptin sensitivity, we have previously reported that leptin, acting in the central nervous system, regulates cardiac lipid metabolism increasing the expression of genes involved in the myocardial intracellular lipolysis (ATGL and HSL) and mitochondrial/peroxisomal fatty acid utilization (PDK4, UCP3, Acox1), whereas decreased those involved in TAG synthesis (SCD-1 and DGAT1), leading these changes to a significant reduction in cardiac TAG and TBARS content [15]. In addition, central leptin markedly increased heart glucose transport independently of insulin. Moreover, the pharmacological inhibition of PPARβ/δ decreased the effects induced by central leptin on the expression of genes involved in lipid metabolism and increased cardiac TAG content [15]. These results support that central leptin plays a critical role in protecting the heart from excess lipid accumulation that, when uncontrolled, can drive cardiac lipotoxicity [7] and align with the suggestion of Baskin and Taegtmeyer [1] who proposed that signals from intermediary metabolism of energy-providing substrates are likely mediators of atrophic remodeling.

Beyond the regulation of cardiac metabolism and protection against cardiac lipotoxicity, we have recently demonstrated that, in adult rats with normal leptin sensitivity, central leptin-induced an atrophy-related gene program in cardiac tissue activating FoxO1/3 [12], master regulators of the atrophy program [42,43], increasing the content of Atrogin-1 and MURF-1, proteins involved in limiting cardiac hypertrophy. Besides, central leptin activated the anti-hypertrophic kinase GSK3β, while the phenotypic markers of cardiac hypertrophy atrial natriuretic peptide (ANP) and β-myosin heavy chain, gene product of Myh7 were decreased in response to central leptin infusion. Finally, the pharmacological inhibition of PPARβ/δ, via in vivo administration of the selective antagonist GSK0660, blunted the induction of FOXO1/3, Atrogin-1, MuRF1, and GSK3β in the heart mediated by central leptin infusion [12].

On the other hand, central leptin inhibited mTORC1 pathway by decreasing protein and basal phosphorylation levels of mTORC1, RAPTOR protein abundance, and basal phosphorylation levels of 4E-BP1 and S6K1. These data may suggest that central leptin modulates the mTORC1 pathway to suppress anabolic processes in the heart [12]. In this sense, it is known that the inhibition of the mTORC-S6K1 pathway regresses established cardiac hypertrophy in mice with transverse aortic constriction [44].

Taken together, the results presented in this study and our prior findings [12,15] suggest that central leptin may exert cardioprotective effects in adult rats with normal leptin sensitivity by facilitating atrophic remodeling, potentially linked with metabolic unloading. This process could imply the induction of FoxO1/3, the inhibition of mTORC1, and the activation of PPARβ/δ pathways.

In addition, this study examines the impact of central leptin infusion and the pharmacological inhibition of PPARβ/δ on cardiac remodeling and cardiac function in Wistar rats. We confirm that leptin may have protective effects against abnormal ventricular growth, as confirmed by MRI, while PPARβ/δ antagonism might lead to changes in cardiac electrical activity indicative of left ventricular hypertrophy (LVH). Our results provide new insights into the physiological mechanisms by which the leptin-PPARβ/δ crosstalk impacts cardiac remodeling and cardiac function in rats.

In contrast, treatment with the PPARβ/δ antagonist GSK0660 resulted in increased QTc duration, indicating potential alterations in ventricular repolarization and an increased risk of arrhythmias. Hence, antagonists like GSK0660 could potentially be used to study metabolic pathways but might also have implications for disrupting energy balance if not used appropriately.

These results underscore the importance of leptin in mediating cardiac remodeling through metabolic pathways. Future research should focus on identifying specific metabolic intermediates and signaling pathways involved in leptin’s cardioprotective effects. Understanding these mechanisms could provide new insights into potential therapeutic strategies for managing cardiac diseases linked with metabolic disorders.

## 4. Conclusions and Future Directions

This study explores the specific interactions between leptin and PPARβ/δ in cardiac function. It was conducted using animal models with normal leptin sensitivity. However, the findings support that inhibition of PPARβ/δ-associated pathways may interfere with central leptin’s beneficial effects in the heart, which may be relevant in patients with conditions like generalized lipodystrophy where leptin therapy is used to mitigate cardiac hypertrophy [45]. Further research is needed to explore the long-term effects of leptin and PPARβ/δ modulation on cardiac health.

## Figures and Tables

**Table 1:** MRI parameters for LVEF acquisition.

Repetition time	8 ms
Echo time	2.5 ms
Slices thickness	1.5 mm
FOV	40 × 40 mm
Matrix	256 × 256
Resolution	0.156 × 0.156

FOV: Field of View.

**Table 2:** Effects of central leptin on hypothalamic and cardiac gene expression levels.

Hypothalamic Gene	SS	PF	LEP
*Ob-Rb*	1.0 ± 0.3 ^a^	2.7 ± 0.2 ^b^	3.7 ± 0.4 ^c^
*Crh*	0.9 ± 0.1 ^a^	0.6 ± 0.1 ^b^	1.96 ± 0.1 ^c^
*Trh*	1.1 ± 0.1 ^a^	0.9 ± 0.1 ^a^	2.6 ± 0.3 ^b^

Hypothalamic mRNA levels of *Ob-Rb*, *Crh,* and *Trh* in leptin (Lep) or saline-infused rats (SS and PF). The values are expressed relative to the SS group. Statistical analysis was performed using one-way ANOVA followed by Tukey’s test. Data are the mean ± SEM (n = 5 per treatment). Different letters indicate significant differences (*p* ≤ 0.05). Groups: SS: saline-infused rats fed *ad libitum*; PF: saline-infused *pair-fed* rats; Lep: leptin-infused rats.

**Table 3:** Effects of central leptin on adipose tissue weights (g) and serum profiles.

	PF + DMSO	PF + GSK0660	LEP + DMSO	LEP + GSK0660
*PrWAT* (g)	4.62 ± 0.86 ^a^	4.86 ± 1 ^a^	3.04 ± 0.85 ^a^	3.71 ± 0.68 ^a^
*eWAT* (g)	4.21 ± 0.36 ^a^	4.80 ± 0.86 ^a^	3.10 ± 0.54 ^a^	3.53 ± 0.40 ^a^
*BAT* (g)	0.42 ± 0.05 ^a^	0.40 ± 0.07 ^a^	0.29 ± 0.02 ^a^	0.39 ± 0.04 ^a^
*Serum glucose* (mg/dL)	99 ± 19 ^a^	105 ± 12 ^a^	112 ± 12 ^a^	118 ± 10 ^a^
*Serum leptin* (ng/mL)	1.07 ± 0.1 ^a^	1.60 ± 0.5 ^a^	1.10 ± 0.3 ^a^	1.14 ± 0.3 ^a^
*Serum insulin* (ng/mL)	2.49 ± 0.8 ^a^	2.67 ± 0.6 ^a^	2.46 ± 0.6 ^a^	5.19 ± 2.1 ^b^

Variations in adipose tissue mass (eWAT, PrWAT and BAT), glucose (mg/dL), leptin (ng/mL) and insulin (ng/mL) values in the serum of animals after 7 days of central administration of leptin plus DMSO, leptin plus GSK0660 or vehicle-infused *pair-fed* rats plus DMSO and vehicle-infused *pair-fed* rats plus GSK0660. Data are the mean ± SEM (n = 5) per group of animals. One-way ANOVA followed by Tukey test (different letters indicate significant differences, *p* ≤ 0.05). Groups: PF+DMSO: vehicle-infused *pair-fed* rats plus DMSO; PF+GSK0660: vehicle-infused *pair-fed* rats plus GSK0660; Lep+DMSO: leptin plus DMSO; Lep+GSK0660: leptin plus GSK0660; PrWAT: peritoneal white adipose tissue; eWAT: epididymal white adipose tissue; BAT: brown adipose tissue.

**Table 4:** ECG signal at (t0) and final time (t7).

	**QRS Duration (ms)**		**∆QRS (ms)**	**R Amplitude (mV)**		**∆R (mV)**
	**t0**	**t7**		**t0**	**t7**	
PF+DMSO	22.7 ± 5.1	19.6 ± 5.1	−3.1 ± 3.2 ^a^	147 ± 19	149 ± 24	2.0 ± 15 ^a^
PF+GSK0660	28.8 ± 5.7	26.3 ± 5.0	−2.5 ± 3.6 ^a^	103 ± 26	95 ± 7	−8 ± 14 ^a^
LEP+DMSO	29.2 ± 7.1	20.4 ± 3.8 *	−8.8 ± 3.6 ^a^	137 ± 23	93 ± 12 *	−44 ± 13 ^b^
LEP+GSK0660	21.4 ± 3.0	17.1 ± 8	−4.3 ± 3.8 ^a^	126 ± 12	132 ± 27	6 ± 13 ^a^
	**QT Duration (ms)**		**∆QT (ms)**	**QTc Duration (ms)**		**∆QTc (ms)**
	**t0**	**t7**		**t0**	**t7**	
PF+DMSO	71.9 ± 13.4	70.9 ± 11.1	−1.0 ± 7.8 ^a^	6.2 ± 1.2	5.6 ± 0.8	−0.6 ± 0.6 ^a^
PF+GSK0660	65.2 ± 7.3	55.1 ± 7.3	−10.1 ± 4.9 ^a,b^	5.4 ± 0.6	4.2 ± 0.5 *	−1.2 ± 0.4 ^a^
LEP+DMSO	79.9 ± 10.5	65.5 ± 12.1	−14.4 ± 7.2 ^b^	6.9 ± 1.0	5.6 ± 1.1	−1.3 ± 0.7 ^a^
LEP+GSK0660	55.3 ± 7.2	60.3 ± 3.6	5.0 ± 3.6 ^a,c^	4.6 ± 0.6	5.1 ± 0.3	0.5 ± 0.3 ^b^

Duration of the QRS wave (ms), the amplitude of the R wave (mV), the duration of the QT interval (ms), and the duration of QTc (QT corrected by Bazett’s formula) of the rats belonging to the four treatment groups at baseline and end time. Values are expressed relative to control rats (PF+DMSO). Results are the mean ± SEM (n = 5) per group of animals. Differences between the two times were assessed using the unpaired Student’s *t*-test (* *p* ≤ 0.05 t7 vs. t0). Differences between treatments were assessed using one-way ANOVA followed by the Tukey test (different letters indicate significant differences, *p* ≤ 0.05). Groups: PF+DMSO: vehicle-infused *pair-fed* rats plus DMSO; PF+GSK0660: vehicle-infused *pair-fed* rats plus GSK0660; Lep+DMSO: leptin plus DMSO; Lep+GSK0660: leptin plus GSK0660.

**Table 5:** Sinus rhythm (R-R interval, R-R) and mean heart rate (HR) for treated groups.

	R-R INTERVAL (ms)		∆R-R (ms)	Heart Rate (bpm)		∆HR (bpm)
	t0	t7		t0	t7	
PF+DMSO	136 ± 6	159 ± 7 *	23 ± 4.1 ^a^	210 ± 22	274 ± 30 *	64 ± 16.64 ^a^
PF+GSK0660	145 ± 4	196 ± 31 *	51 ± 14.0 ^b^	205 ± 12	275 ± 34 *	70 ± 16.12 ^a^
LEP+DMSO	135 ± 5	139 ± 4	4 ± 2.9 ^c^	231 ± 30	219 ± 20	−12 ± 16.12 ^b^
LEP+GSK0660	141 ± 4	142 ± 6	1 ± 3.2 ^c^	199 ± 4	224 ± 14 *	25 ± 6.51 ^c^

Sinus rhythm (R-R), measured in milliseconds (ms), and heart rate (HR), measured in beats per minute (bpm) of the rats belonging to the four treatment groups at baseline and final time. Values are expressed relative to control rats (PF+DMSO). Results are the mean ± SEM (n = 5) per group of animals. One-way ANOVA followed by Tukey test (different letters indicate significant differences, *p* ≤ 0.05). Differences between the two times were assessed using the unpaired Student’s *t*-test (* *p* ≤ 0.05 t7 vs. t0). Differences between treatments were assessed using one-way ANOVA followed by the Tukey test (different letters indicate significant differences, *p* ≤ 0.05). Groups: PF+DMSO: vehicle-infused *pair-fed* rats plus DMSO; PF+GSK0660: vehicle-infused *pair-fed* rats plus GSK0660; Lep+DMSO: leptin plus DMSO; Lep+GSK0660: leptin plus GSK0660.

## Data Availability

Data will be made available on request.

## Supplementary Materials

The following supporting information can be downloaded at https://www.mdpi.com/article/10.3390/biom14081028/s1; Table S1: Primers sequences of genes used for quantification of mRNAs by qRT-PCR; Figure S1: Representative Red Ponceau staining of the nitrocellulose membrane for total protein normalization prior to immunodetection; Table S2: Quantitative densitometry analysis of PPARβ/δ protein bands from the Western-Blot.
