# Supplementary material for: Central Actions of Leptin Induce an Atrophic Pattern and Improves Heart Function in Lean Normoleptinemic Rats via PPARβ/δ Activation
Source: Biomolecules. 2024 Aug 18;14(8):1028. doi: 10.3390/biom14081028 (PMC11352611; doi:10.3390/biom14081028)
Supplement: Supplementary file 1 [file biomolecules-14-01028-s001.zip › biomolecules-3095065-supplementary.pdf]

Central actions of leptin induce an atrophic pattern and improves heart function in lean normoleptinemic rats via PPARβ/δ activation

Blanca Rubio, Cristina Pintado, Lorena Mazuecos, Marina Benito, Antonio Andrés and Nilda Gallardo

Supplementary Data

Table S1. Primer sequences of genes used for quantification of mRNAs by qRT-PCR

GENE	NAME	REFERENCE
Ob-Rb	Long Form of Leptin Receptor	Rn01433205_m1
Crh	Corticotropin Releasing Hormone	Rn01462137_m1
**NAME**	**SEQUENCE**	
Trh_F	AGCTCAGCATCTTGGAAAGC	
Trh_R	CCAGCAGCAACCAAGGTC	

a **b**

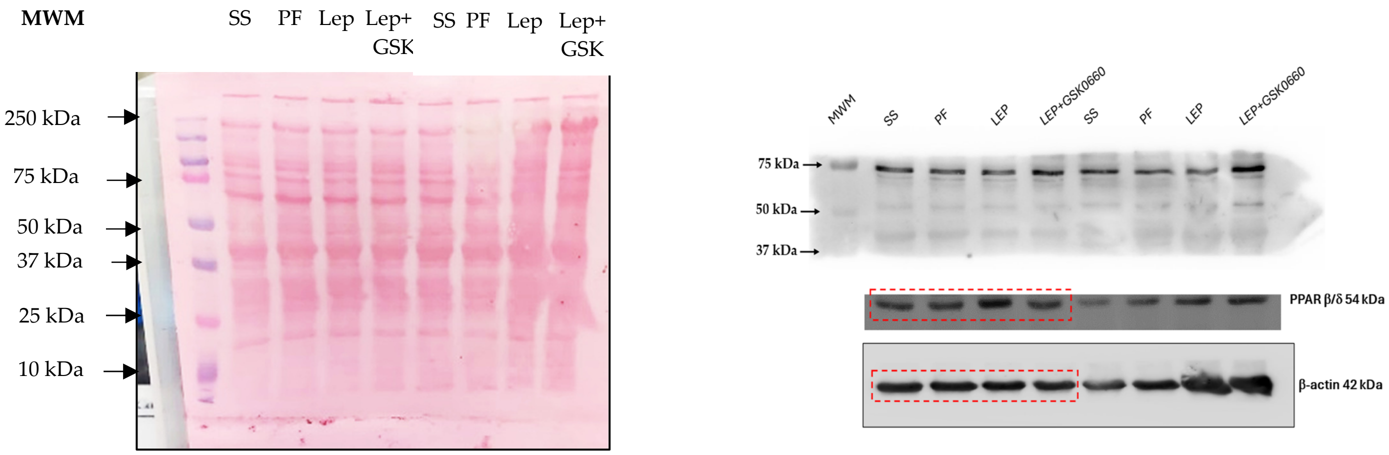

Figure S1: **(a)** Red Ponceau staining of the nitrocellulose membrane for total protein normalization after transferring and **(b)** uncropped images immunodetection correspond-ing to Figure 2. Membranes were cut with a scalpel to allow concomitant detection of

more than one protein on the same membrane. Bands used in Figure 2 are marked by dashed red boxes. Molecular weight markers (MWM arrows).

Equal amounts of proteins from cardiac ventricles total extracts (50 µg) from vehicle (SS, PF), leptin, and leptin plus the antagonist GSK0660 (treated rats were separated under reducing conditions on a 10% SDS-PAGE gel and further stained with Red Ponceau tinction before incubation with PPARβ/δ antibody (1:1000, ab23676) and β-actin antibody (1:1000, ab8226).

Table S2. Quantitative densitometry analysis of PPARβ/δ protein bands from the Western Blot. The images were subjected to a densitometric analysis with a G-Box Densitometer. Bands were quantified by scanning densitometry with the exposure in the linear range using Gene Tools software (Syngene, Cambridge, UK) and the protein levels were expressed in arbitrary units.

Treatment	PPARβ/δ Raw values	β-actin Raw values	Ratio PPARβ/δ/β-actin	Relative Units (Mean SS as control, unit 1)
**SS**	351963264	249088848	1,4130029	1,109032602
**PF**	353496608	239901568	1,47350687	1,156520739
**LEP**	534628746	253160944	2,11181368	1,657512684
**LEP+GSK0660**	368204320	275426624	1,33685086	1,049262668
**SS**	344023360	303059136	1,13516908	0,890967398
**PF**	346664032	251347328	1,37922306	1,082519608
**LEP**	484653312	248088356	1,95355123	1,533296217
**LEP+GSK0660**	343133440	237857977	1,44259799	1,132261092
